# Supplementary material for: Utilizing AgNPt-SALDI to Classify Edible Oils by Multivariate Statistics of Triacylglycerol Profile
Source: Molecules. 2021 Sep 28;26(19):5880. doi: 10.3390/molecules26195880 (PMC8510378; doi:10.3390/molecules26195880)
Supplement: Supplementary file 1 [file molecules-26-05880-s001.zip › molecules-1386969-supplementary.pdf]

Supporting Information of

Utilizing AgNPt-SLADI to Classify Edible Oils by Multivariate Statistics of Triacylglycerol Profile

Tzu-Ling Yang,[1] Cheng-Liang Huang,[1] Chuping Lee[2,*]

[1]Department of Applied Chemistry, National Chiayi University, Chiayi City 60004, Taiwan
[2]Department of Chemistry, Fu Jen Catholic University, New Taipei City 24205, Taiwan

*To whom correspondence should be addressed: 152305@gapp.fju.edu.tw

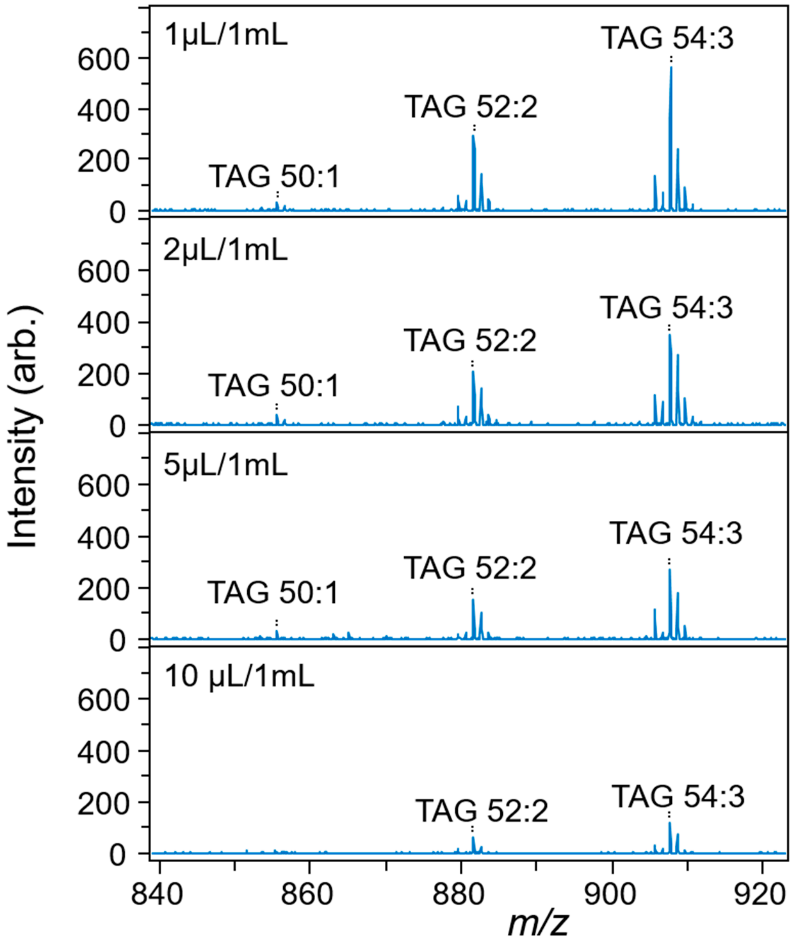

Figure S1. Mass spectra of TAG profiles obtained from samples prepared using AgNPts in the positive ion mode at various concentrations (1, 2, 5, and 10 μLmL^{-1}). Each spectrum was acquired by summing those from 200 laser shots.

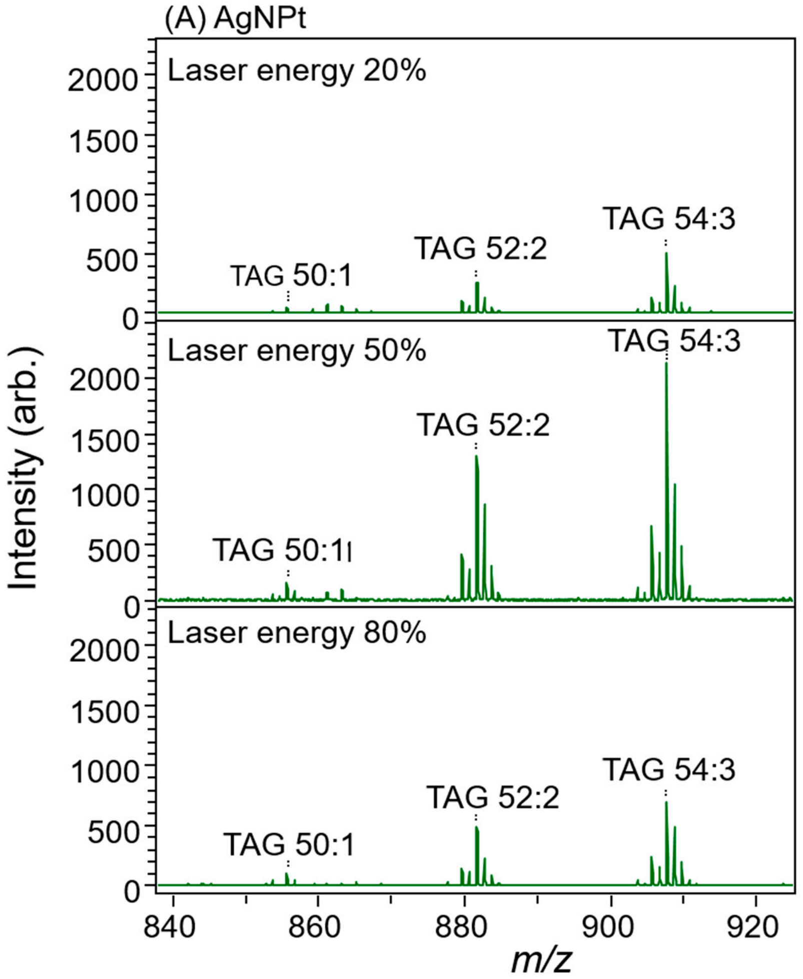
(A) AgNPt
Laser energy 20%
Laser energy 50%
Laser energy 80%
TAG 50:1
TAG 52:2
TAG 54:3
TAG 50:1
TAG 52:2
TAG 54:3
TAG 50:1
TAG 52:2
TAG 54:3
Intensity (arb.)
0
500
1000
1500
2000
840
860
880
900
920
m/z

Figure S2.

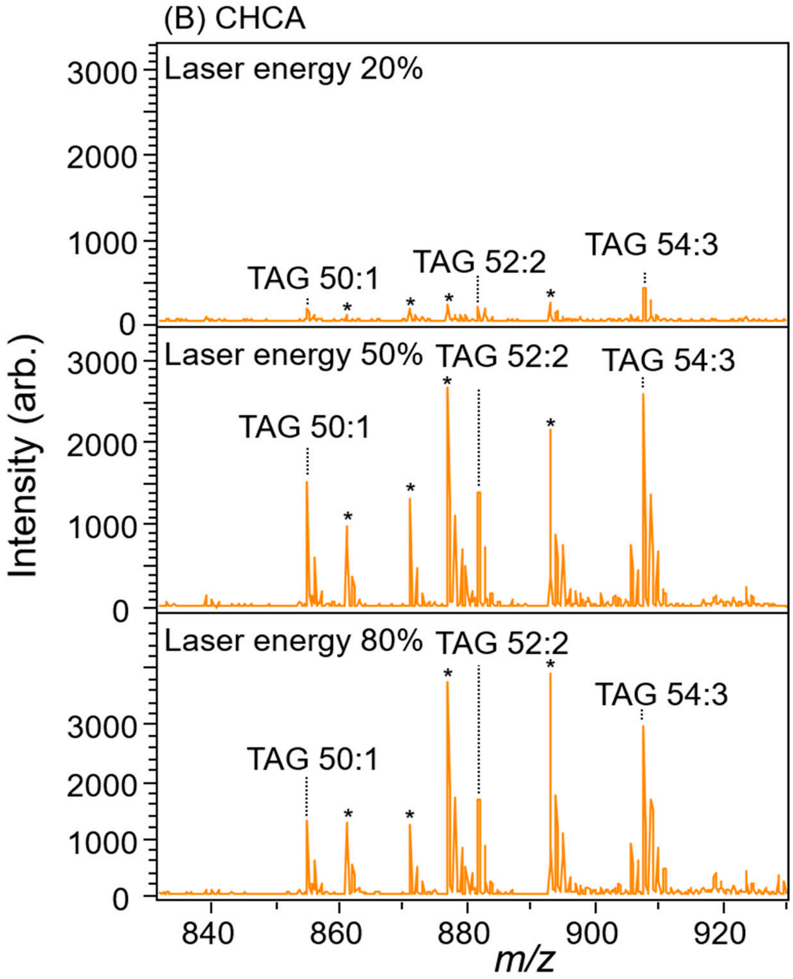
(B) CHCA
Laser energy 20%
Laser energy 50%
Laser energy 80%
TAG 50:1
TAG 52:2
TAG 54:3
Intensity (arb.)
m/z
0
1000
2000
3000
840
860
880
900
920

Figure S2.

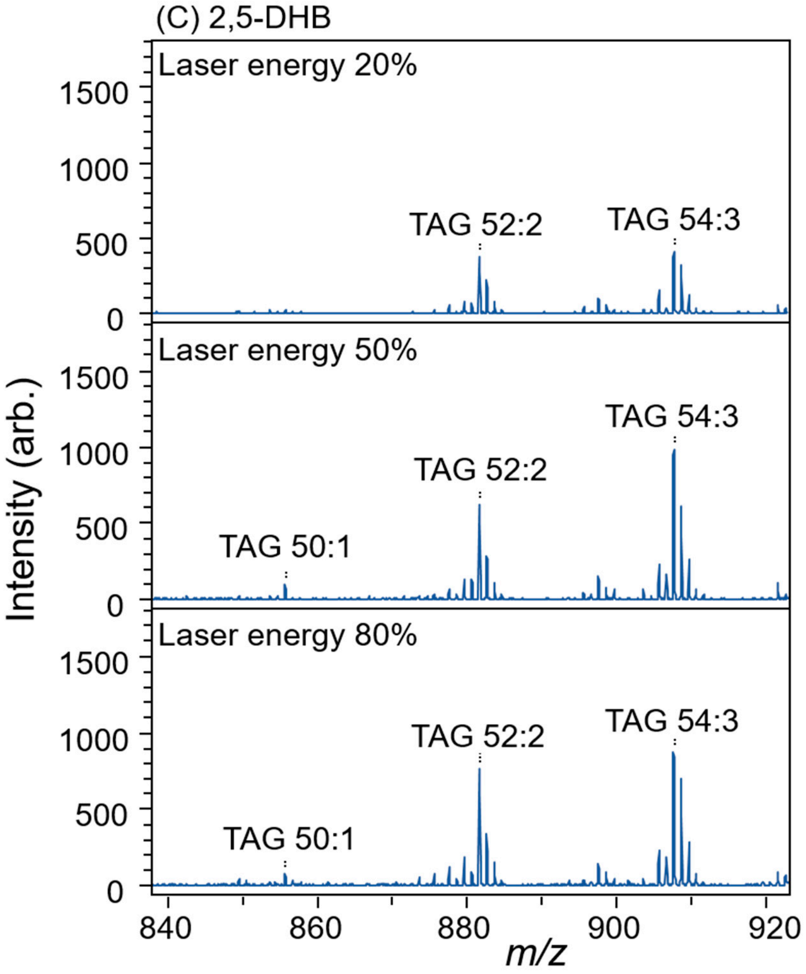

Figure S2. Mass spectra of TAG profiles obtained from samples prepared using (A) AgNPts, (B) CHCA, and (C) 2,5-DHB in the positive ion mode at 30%, 50%, and 80% laser energy. Each spectrum was acquired by those from summing 200 laser shots.

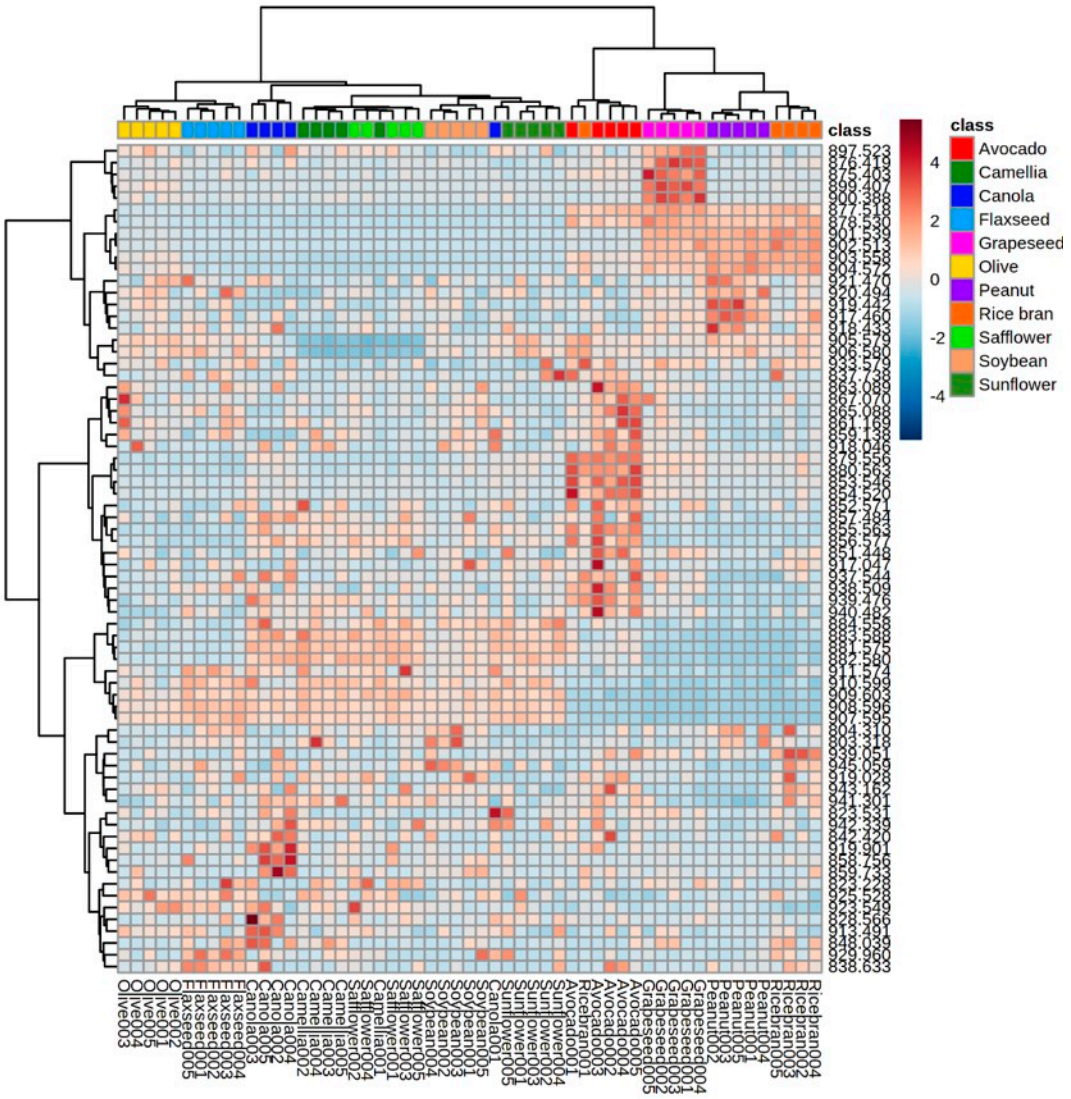

Figure S3. Heatmap visualization constructed using 70 TAG features. Rows represent TAG features; columns represent samples prepared using the thin layer method and AgNPts. Dark red indicates a high level; dark blue indicates a low level.

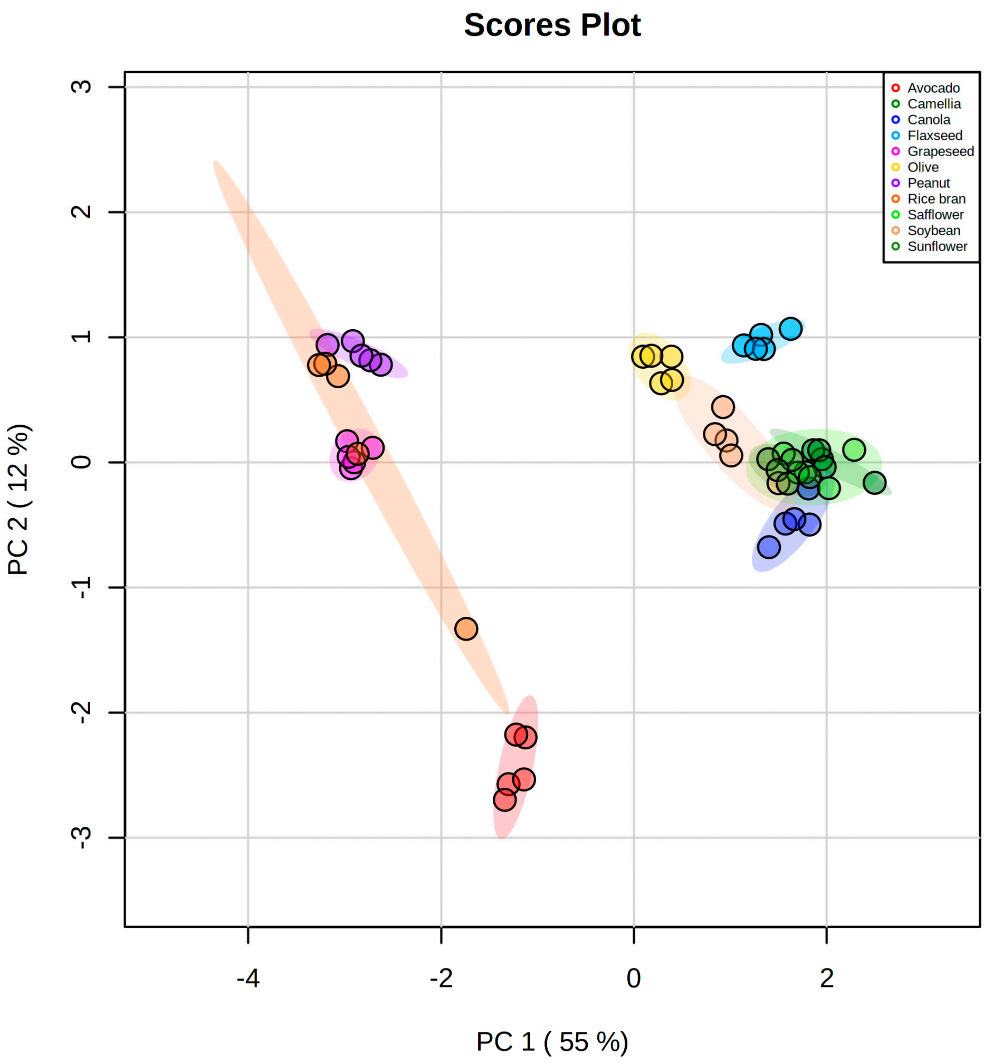

Figure S4. PCA score plot for eleven edible oils, based on the SALDI MS results, with samples prepared using the thin layer method and AgNPts. The first and second principal components accounted for 55.0% and 12.0% of the variance, respectively.

(A)

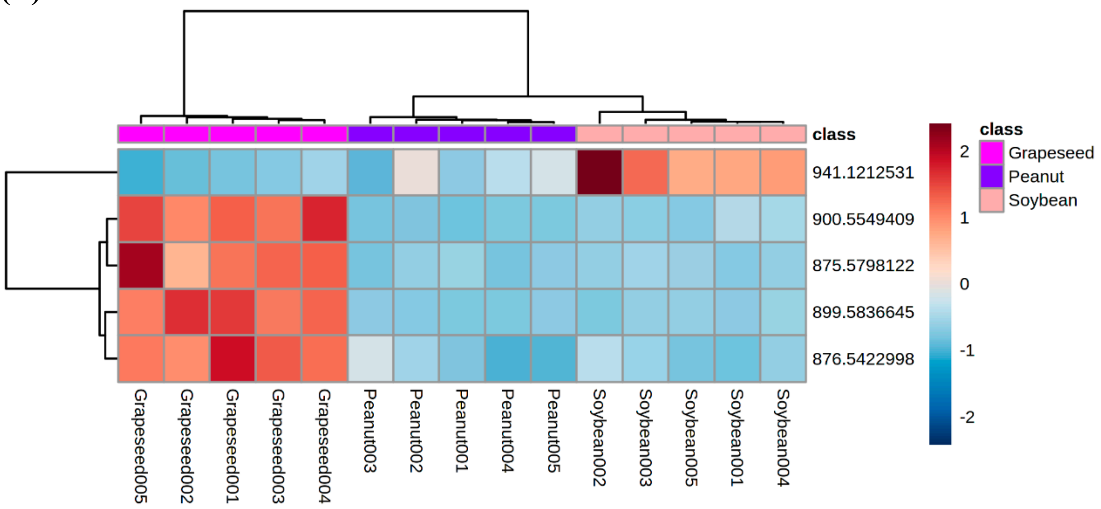

(B)

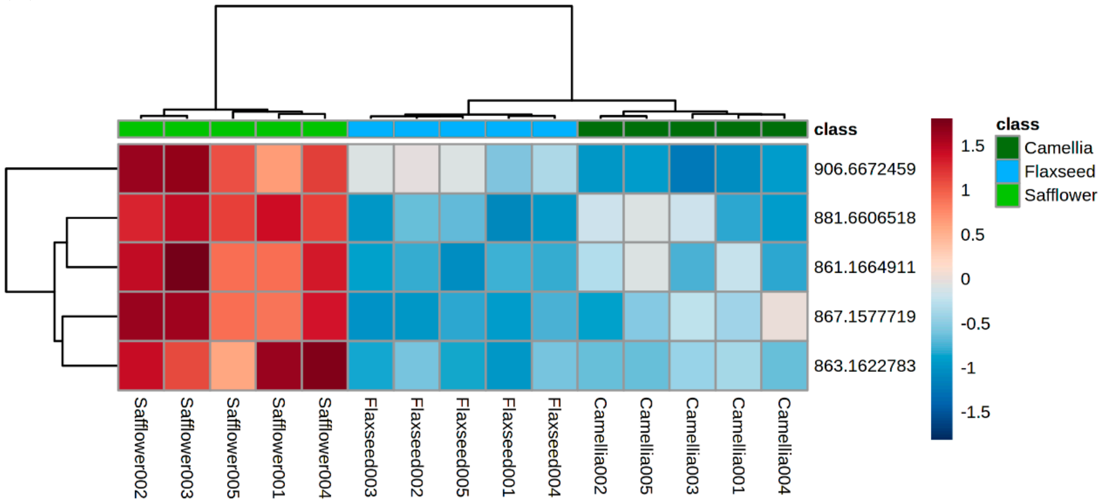

Figure S5. Heatmap visualization constructed using five TAG features. Rows represent TAG features; columns represent samples: (A) group 1 and (B) group 2. Dark red indicates a high level; dark blue indicates a low level.

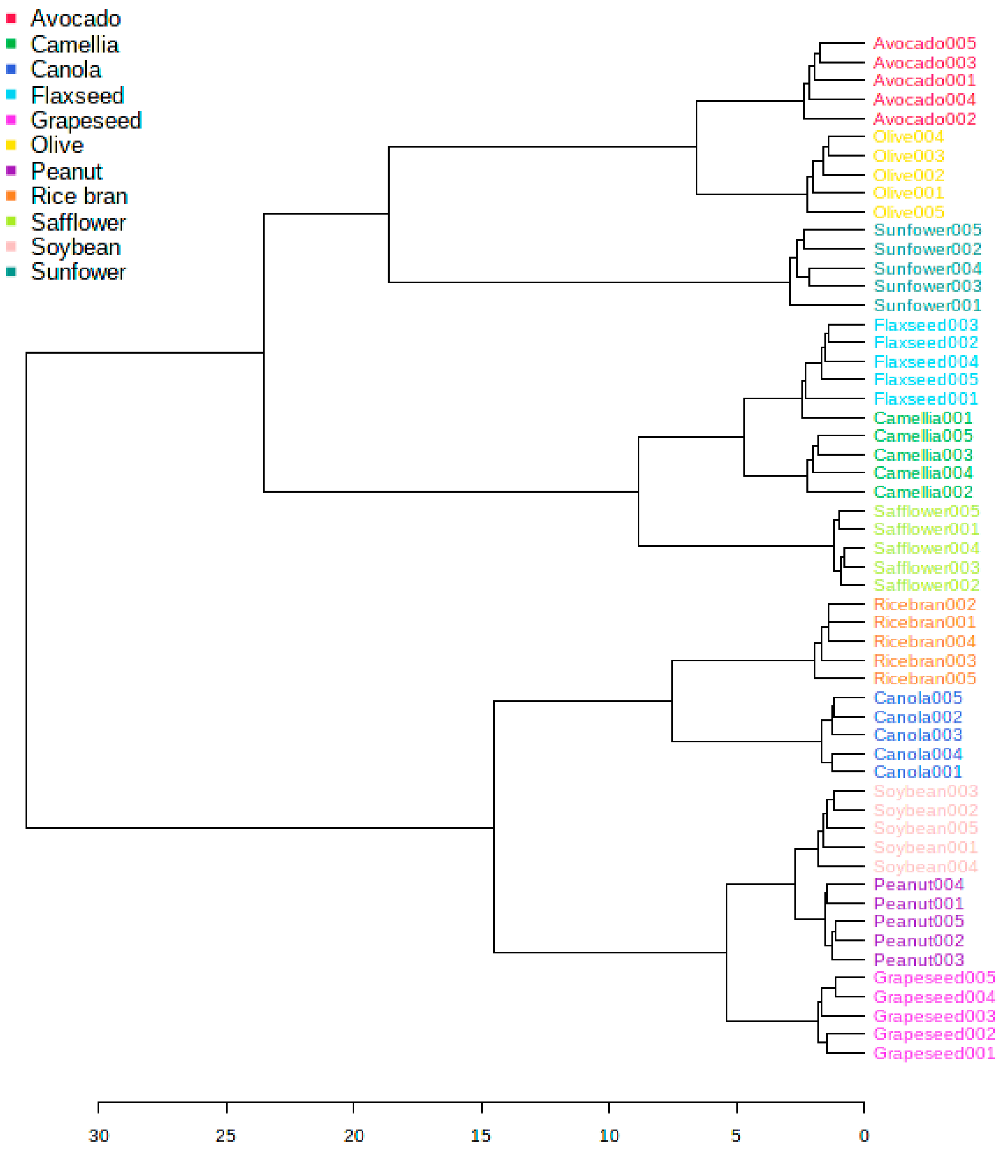

Figure S6. Hierarchical clustering result for eleven edible oils, based on the SALDI MS results.

Table S1. Summary of major TAGs signals in the eleven edible oils.

Oil	TAG	ion	*m/z*
Olive	50:1	$[M+Na]^+$	855.6
	52:3	$[M+Na]^+$	879.7
	52:2	$[M+Na]^+$	881.7
	54:4	$[M+Na]^+$	905.7
	54:3	$[M+Na]^+$	907.7
	54:2	$[M+Na]^+$	909.7
Camellia	50:2	$[M+Na]^+$	853.7
	50:1	$[M+Na]^+$	855.6
	52:4	$[M+Na]^+$	877.7
	52:3	$[M+Na]^+$	879.7
	52:2	$[M+Na]^+$	881.7
	54:7	$[M+Na]^+$	899.6
	54:6	$[M+Na]^+$	901.7
	54:5	$[M+Na]^+$	903.7
	54:4	$[M+Na]^+$	905.7
	54:3	$[M+Na]^+$	907.7
Canola	50:2	$[M+Na]^+$	853.6
	52:4	$[M+Na]^+$	877.7
	52:3	$[M+Na]^+$	879.7
	52:2	$[M+Na]^+$	881.7
	54:7	$[M+Na]^+$	899.7
	54:6	$[M+Na]^+$	901.7
	54:5	$[M+Na]^+$	903.7
	54:4	$[M+Na]^+$	905.7
	54:3	$[M+Na]^+$	907.7
Sunflower	52:4	$[M+Na]^+$	877.6
	52:3	$[M+Na]^+$	879.7
	52:2	$[M+Na]^+$	881.7
	54:6	$[M+Na]^+$	901.7
	54:5	$[M+Na]^+$	903.7
	54:4	$[M+Na]^+$	905.7
	54:3	$[M+Na]^+$	907.7
Soybean	50:2	$[M+Na]^+$	853.6
	52:4	$[M+Na]^+$	877.7
	52:3	$[M+Na]^+$	879.7
	52:2	$[M+Na]^+$	881.7
	54:7	$[M+Na]^+$	899.6
	54:6	$[M+Na]^+$	901.7
	54:5	$[M+Na]^+$	903.7
	54:4	$[M+Na]^+$	905.7
	54:3	$[M+Na]^+$	907.7
Safflower	52:3	$[M+Na]^+$	879.7
	52:2	$[M+Na]^+$	881.7
	54:5	$[M+Na]^+$	903.7
	54:4	$[M+Na]^+$	905.7
	54:3	$[M+Na]^+$	907.7
Rice bran	50:2	$[M+Na]^+$	853.6
	50:1	$[M+Na]^+$	855.5
	52:4	$[M+Na]^+$	877.5
	52:3	$[M+Na]^+$	879.7
	52:2	$[M+Na]^+$	881.7
	54:6	$[M+Na]^+$	901.7

	54:5	$[M+Na]^+$	903.7
	54:4	$[M+Na]^+$	905.7
	54:3	$[M+Na]^+$	907.7
Avocado	50:3	$[M+Na]^+$	851.7
	50:2	$[M+Na]^+$	853.6
	52:1	$[M+Na]^+$	855.6
	52:4	$[M+Na]^+$	877.7
	52:3	$[M+Na]^+$	879.7
	52:2	$[M+Na]^+$	881.7
	54:5	$[M+Na]^+$	903.7
	54:4	$[M+Na]^+$	905.7
	54:3	$[M+Na]^+$	907.7
Flaxseed	52:6	$[M+Na]^+$	873.7
	52:5	$[M+Na]^+$	875.7
	52:4	$[M+Na]^+$	877.7
	52:3	$[M+Na]^+$	879.7
	52:2	$[M+Na]^+$	881.7
	54:9	$[M+Na]^+$	895.7
	54:8	$[M+Na]^+$	897.6
	54:7	$[M+Na]^+$	899.6
	54:6	$[M+Na]^+$	901.7
	54:5	$[M+Na]^+$	903.7
	54:4	$[M+Na]^+$	905.7
	54:3	$[M+Na]^+$	907.7
Grapeseed	50:2	$[M+Na]^+$	855.6
	52:4	$[M+Na]^+$	877.8
	52:3	$[M+Na]^+$	879.7
	52:2	$[M+Na]^+$	881.7
	54:6	$[M+Na]^+$	901.7
	54:5	$[M+Na]^+$	903.7
	54:4	$[M+Na]^+$	905.7
	54:3	$[M+Na]^+$	907.7
Peanut	50:2	$[M+Na]^+$	853.6
	50:1	$[M+Na]^+$	855.6
	52:4	$[M+Na]^+$	877.8
	52:3	$[M+Na]^+$	879.7
	52:2	$[M+Na]^+$	881.7
	54:6	$[M+Na]^+$	901.7
	54:5	$[M+Na]^+$	903.7
	54:4	$[M+Na]^+$	905.7
	54:3	$[M+Na]^+$	907.7

Table S2. Annotations of TAG species in the SALDI MS of edible oils.

TAG species	Ion formula	Cal. *m/z*	Exp. *m/z*
50:2	$[C_{53}H_{98}O_6+Na]^+$	853.7	853.7
50:1	$[C_{53}H_{100}O_6+Na]^+$	855.7	855.7
52:6	$[C_{55}H_{94}O_6+Na]^+$	873.7	873.7
52:5	$[C_{55}H_{96}O_6+Na]^+$	875.7	875.7
52:4	$[C_{55}H_{98}O_6+Na]^+$	877.7	877.7
52:3	$[C_{55}H_{100}O_6+Na]^+$	879.7	879.8
52:2	$[C_{55}H_{102}O_6+Na]^+$	881.7	881.7
54:9	$[C_{57}H_{92}O_6+Na]^+$	895.7	895.6
54:8	$[C_{57}H_{94}O_6+Na]^+$	897.7	897.7
54:7	$[C_{57}H_{96}O_6+Na]^+$	899.7	899.7
54:6	$[C_{57}H_{98}O_6+Na]^+$	901.7	901.7
54:5	$[C_{57}H_{100}O_6+Na]^+$	903.7	903.7
54:4	$[C_{57}H_{102}O_6+Na]^+$	905.7	905.7
54:3	$[C_{57}H_{104}O_6+Na]^+$	907.7	907.7
54:2	$[C_{57}H_{106}O_6+Na]^+$	909.7	909.7
